# Understanding Strain‐Induced Phase Transformations in BiFeO_3_ Thin Films

**DOI:** 10.1002/advs.201500041

**Published:** 2015-05-28

**Authors:** Hemant Dixit, Christianne Beekman, Christian M. Schlepütz, Wolter Siemons, Yongsoo Yang, Nancy Senabulya, Roy Clarke, Miaofang Chi, Hans M. Christen, Valentino R. Cooper

**Affiliations:** ^1^ Materials Science and Technology DivisionOak Ridge National LabOak RidgeTN37831USA; ^2^ Advanced Photon SourceArgonne National LaboratoryArgonneIL60439USA; ^3^ Department of PhysicsUniversity of MichiganAnn ArborMI48109USA; ^4^ Center for Nanophase Materials SciencesOak Ridge National LabOak RidgeTN37830USA

**Keywords:** multiferroic BiFeO_3_, phase coexistence, piezoelectric response, solid‐state nudged elastic band method

## Abstract

Experiments demonstrate that under large epitaxial strain a coexisting striped phase emerges in BiFeO_3_ thin films, which comprises a tetragonal‐like (*T*′) and an intermediate *S*′ polymorph. It exhibits a relatively large piezoelectric response when switching between the coexisting phase and a uniform *T*′ phase. This strain‐induced phase transformation is investigated through a synergistic combination of first‐principles theory and experiments. The results show that the *S*′ phase is energetically very close to the *T*′ phase, but is structurally similar to the bulk rhombohedral (*R*) phase. By fully characterizing the intermediate *S*′ polymorph, it is demonstrated that the flat energy landscape resulting in the absence of an energy barrier between the *T*′ and *S*′ phases fosters the above‐mentioned reversible phase transformation. This ability to readily transform between the *S*′ and *T*′ polymorphs, which have very different octahedral rotation patterns and *c*/*a* ratios, is crucial to the enhanced piezoelectricity in strained BiFeO_3_ films. Additionally, a blueshift in the band gap when moving from *R* to *S*′ to *T*′ is observed. These results emphasize the importance of strain engineering for tuning electromechanical responses or, creating unique energy harvesting photonic structures, in oxide thin film architectures.

## 1 Introduction

Piezoelectric perovskites have a wide range of technological applications, including sonar devices and piezoelectric fuel injectors in automobile engines. The lead‐based Pb(Zr*_x_*Ti_1_ *_−x_*)O_3_ has remained the prototypical piezoelectric due to its high electromechanical response near the composition‐dependent morphotropic phase boundary (MPB). However, the growing concern regarding the toxicity of lead‐containing devices has prompted the search for an environmentally friendly alternative. Bismuth ferrite, which is a ferroelectric material with a Curie temperature of 1100 K^1^ and rhombohedral symmetry (space group *R*3*c*) in its ground state,^2^, ^3^ is an appropriate choice. The lone pair (s^2^ orbital) on the Bi cation plays an important role in driving the spontaneous polarization^4^ that varies between ≈1 C m^−2^ for the rhombohedral^5^ phase to a predicted 1.50 C m^−2^ for the strain‐stabilized pseudotetragonal phase,^6^ one of the highest among perovskite structures. Experimentally, it has been shown that strain can be used to induce a morphotropic phase transition in BiFeO_3_ thin films^7^, ^8^, ^9^, ^10^, ^11^ with a structural transformation from the rhombohedral phase to a monoclinic pseudotetragonal (*T*′) phase at compressive epitaxial strains exceeding ≈4.5%. Most strikingly, the *T*′ phase is not structurally uniform but rather a mixed phase exhibiting stripe patterns with a larger piezoresponse than the pure *R* and *T*′ phases, which is believed to be a consequence of the phase interconversion in an applied electric field.^12^

While early work ascribed the stripe patterns to a coexistence between the *T*′ and *R* regions,^7^ it is now known that an intermediary polymorph, referred to as the *S*′ phase, forms with a *c*‐axis lattice parameter close to the midpoint between the *R* and *T*′ values.^13^, ^14^ Note the highly strained BiFeO_3_ thin films, grown on a LaAlO_3_ substrate, only show coexisting *T*′ and *S*′ phases and an absence of any *R*‐like region. Although the *R* and *T*′ phases have been extensively studied, the novel polymorph, i.e., the *S*′ phase, has not been well characterized even though it has become apparent that its existence is the key to the intriguing piezoelectric and ferroelectric properties.^13^ In this article, we study these phases and their coexistence to reveal the mechanisms behind the strain‐induced transitions in structural, electronic, and ferroelectric properties in epitaxial BiFeO_3_ thin films.

BiFeO_3_ films are modeled using the experimental unit cell parameters determined from X‐ray diffraction (XRD) studies on the *T*′ and *S*′ phases. We find that while the *S*′ phase is energetically close to the *T*′ phase, it shows similar out‐of‐phase FeO_6_ octahedra rotational patterns as the *R* phase. The optimized structure for the *T*′ phase exhibits a zig‐zag arrangement of the Bi cations along the lattice vectors, while they are symmetric in the *S*′ and *R* structures. We also find that the differences in the calculated XRD half‐order peaks of the *T*′ and *S*′ structures are in agreement with those obtained from experiment for the coexisting phases (The term “half‐order peak” is used here in the conventional way to refer to peaks that would be absent if the unit cell contained only one formula unit of *AB*O_3_, but can be indexed as half‐integer Bragg peaks corresponding to such a unit cell). These half‐order peaks are highly sensitive to such Bi displacements and thus do not correspond only to the octahedral rotational patterns, in contrast to many other perovskites. Furthermore, we calculate a blueshift in the band gap (*E*_g_) as a function of applied compressive strain. The calculated blueshift between the *S*′ and *T*′ phases is experimentally confirmed by electron energy loss spectroscopy (EELS). Finally, the flat energy landscape as established by the minimum energy path between the *T*′ and *S*′ polymorphs explains the experimentally observed reversible phase transformation under the application of an electric field between the two. The high piezoelectric response near the MPB can thus be attributed to the presence of the coexisting phases with similar energies and dramatically different lattice parameters resulting from changes in octahedral rotation patterns.

## 2 Results

### 2.1 Structural Properties

Previous theoretical studies have observed a large number of potentially stable phases for the *T*′ phase.^15^ Thus, in order to most closely simulate experimental conditions, we optimize the total energy by imposing the experimental geometries. To this end, we first perform careful structural characterization of the films with coexisting *T*′ and *S*′ structures using synchrotron XRD. Data collection and analysis is complicated by the well‐known fact that the *S*′ polymorph exists in domains that are both tilted (around an inplane rotation axis) and twisted (around an axis perpendicular to the film surface) with respect to the substrate, i.e., the striped patterns are comprised of these tilted *S*′ and tilted *T*′ domains.^13^, ^16^ This requires the acquisition of 3D maps through reciprocal space. From such maps, and a least‐squares refinement, the unit cell parameters of the triclinic *S*′ structure are found.

**Figure 1:**
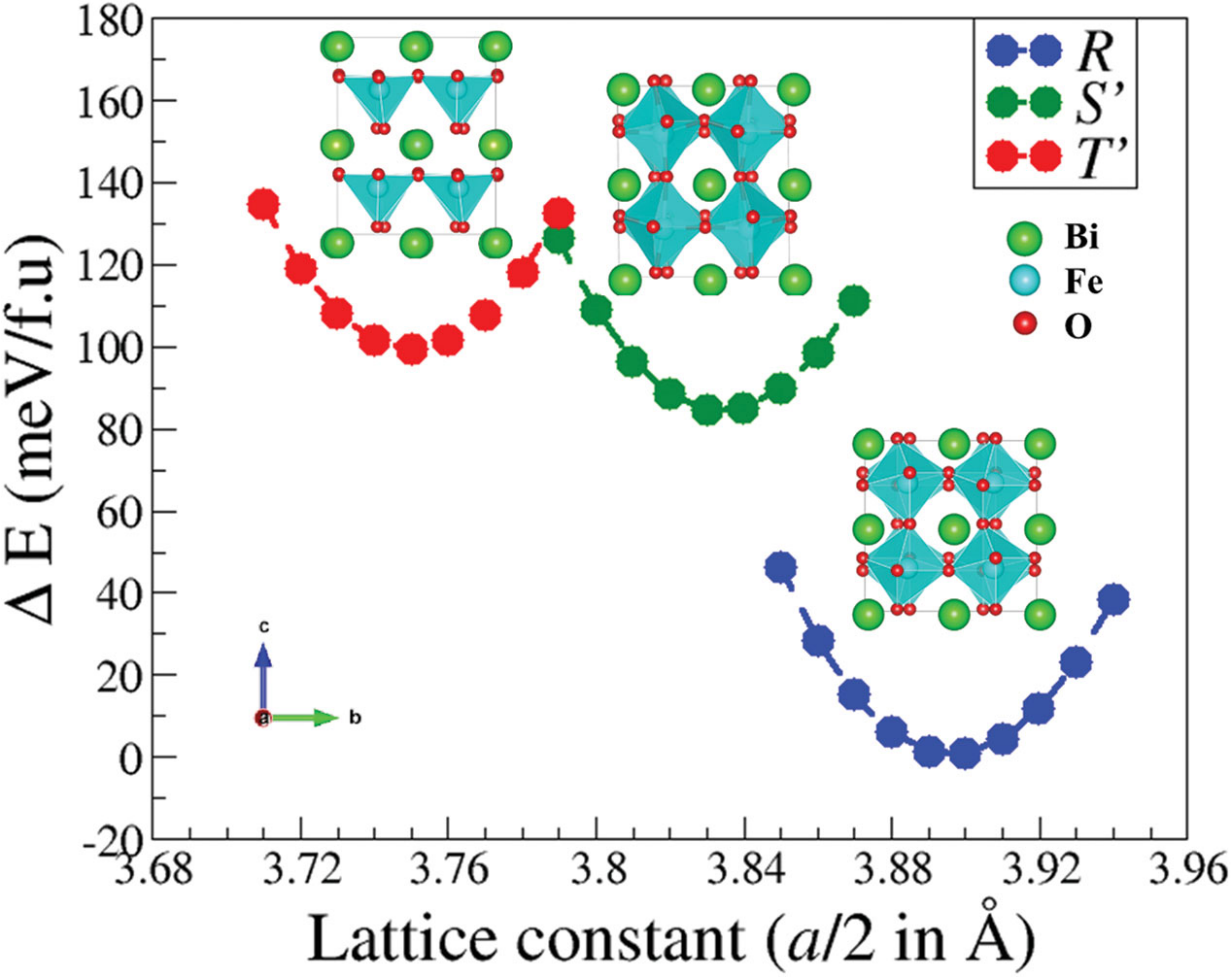
Total energy curves as a function of lattice constant (*a*/2) for the *T*′, *S*′, and *R* phases of BiFeO_3_. The *R* phase is the ground state and is used as a reference to plot the energy difference between the *T*′, *S*′, and *R* phases. The Bi, Fe, and oxygen atoms are shown in green, cyan, and red colors, respectively. The images of the optimized structures are generated using VESTA.^37^

Calculations are then performed by fixing the *b*/*a* and *c*/*a* ratios along with the *α*, *β*, and *γ* angles as measured in the experiment. The number of degrees of freedom is then reduced to a single variable—the lattice constant *a*. This also allows us to discuss all aspects of this work without having to scale our DFT results to experimental lattice parameters, despite the fact that it is well known that LDA results will underestimate the actual lattice parameters. The optimized lattice constants are listed in **Table** **1** and the total energy curves along with the optimized structures are shown in **Figure** **1**. The *R* and *T*′ phases serve as benchmarks and can be compared with earlier reports. Consistent with previous theory, we find the *R* phase to be the ground state of BiFeO_3_ with a total energy 84 and 96 meV f.u.^−1^ lower than the *S*′ and *T*′ phases, respectively. The optimized lattice constant (*a* = *b* = *c* = 7.80 Å) for the *R* phase is in excellent agreement with experiment and previous theory.^6^ The rhombohedral phase has *a* ^−^ *a* ^−^ *a* ^−^ rotations of the FeO_6_ octahedra in terms of Glazer notation.

**Table 1:** Optimized (experimental) structural parameters corresponding to the minimum energy configuration, space group, and Glazer tilt patterns for the three phases of BiFeO_3_ considered

Phase	*a* [Å]	*b*/*a*	*c*/*a*	*α*	*β*	*γ*	Space group	Tilt pattern
*R*	7.80 (7.92)	1	1	89.38	89.38	89.38	161 (*R3c)*	*a* ^−^ *a* ^−^ *a* ^−^
*S*′	7.60 (7.82)	0.9744	1.0918	90.51	89.38	90.83	1 (*P*_1_ *)*	*a* ^−^ *b* ^−^ *c* ^−^
*T*′	7.50 (7.62)	0.9790	1.2257	90	88.1	90	1 (*P*_1_ *)*	*a* ^−^ *b* ^−^ *c* ^0^

The optimized lattice constant, *a*, for the *T*′ phase is 7.50 Å, where we impose the experimentally determined unit cell parameters that can be described as corresponding to a shear distortion along the [100] direction. At large compressive strains, the octahedral rotations along the *c*‐axis are completely frozen and an *a* ^−^ *b* ^−^ *c* ^0^ tilt pattern is obtained for the optimized structure. Contrary to previous calculations that only imposed an inplane strain,^6^, ^8^ when we impose the experimentally observed structural parameters (*b*/*a* ratio and shear angle *β*), we retain a lower *P*_1_ symmetry than the previously reported predictions of *Cc* or *Pm* phases.^8^, ^17^, ^18^ This space group determination was performed using the FINDSYM^19^ software. The optimized *P*_1_ geometry, however, is in close energetic proximity with the other, *Cc* or *Pm,* phases with the energy difference between these structures being ≈4 meV f.u.^−1^ 8 The supertetragonal *P4mm* phase lies much higher in energy (≈100 meV f.u.^−1^) compared to the *T*′ phase, whereas the monoclinic *Cm* phase is also few tens of meV away from the *T*′ phase.^15^ It is also interesting to note that the large strain is accompanied by off‐center displacements of the Bi and Fe atoms from their equilibrium positions; resulting in a zig‐zag arrangement of the cations along the direction of the lattice vectors.

The optimized lattice constant, *a*, for the triclinic *S*′ phase is 7.60 Å. The structure has a symmetric Bi cation arrangement and an *a* ^−^ *b* ^−^ *c* ^−^ tilt pattern similar to that of the *R* phase, although the octahedral rotations along the [111] direction are now significantly reduced as a consequence of the biaxial strain. Despite its structural similarity with the *R* phase, the *S*′ phase is energetically very close to the *T*′ phase with an energy difference of 12 meV f.u.^−1^. It should also be noted that although the optimized lattice constants are underestimated with respect to experiment (a consequence of using the LSDA), they have a very similar scaling factor of 1.016 ± 0.02 for all three phases.

Since the predicted structures contain information about distortions within the unit cell, we can compare them directly to the synchrotron XRD data for the half‐order peaks. For this purpose, the X‐ray intensities of the DFT optimized structures were calculated using the GDIS^20^ software (see the Supporting Information for details). In other words, we calculate the X‐ray intensities directly from the atomic positions, not from symmetries determined by FINDSYM or any other analysis. For experiments, we note that unlike a powder pattern, a complete survey of all diffraction peaks is not feasible in an epitaxial film, and the determination of relative intensities is complicated due to the experimental geometry that differs for each peak. We further need to consider that only a small sample fraction contributes to the scattering from the *S*′ phase in a particular direction due to the complex misalignment of these domains. However, a relatively quick survey of a number of peaks yields valuable information. We find that peaks of the type (half‐integer, half‐integer, half‐integer) are present and strong for the *S*′ phase: computation predicts them to have a scatter intensity of about 10^−3^ of the strongest (integer, integer, integer) peaks, and we indeed observe them for (1/2 3/2 1/2), (1/2 1/2 3/2), (1/2 3/2 3/2), (3/2 3/2 3/2), (1/2 1/2 5/2), and (3/2 3/2 5/2). For the *T*′ phase, computation predicts them to exhibit an intensity of only about 10^−7^ of the strongest (integer, integer, integer) peaks. Thus, our observation that they are experimentally detected but weak agrees with the calculations, considering the large volume fraction of the sample in the *T*′ phase, which makes weaker peaks more readily observable. For the (half‐integer, integer, half‐integer) peaks, our computational results predict them to be of comparable intensity for the *T*′ and *S*′ phases [≈10^−6^ of the intensity of the strongest (integer, integer, integer) peaks]. We confirm this experimentally by noting that the (1/2 1 1/2), (1/2 1 3/2), (3/2 1 3/2), (3/2 1 5/2), and (3/2 2 3/2) peaks are observed for the *T*′ majority phase but remain undetected for the *S*′ domains (i.e., the tilted, smaller volume fraction). These comparisons between synchrotron XRD data and computational predictions indicate that the theoretically predicted structures are indeed consistent with those experimentally observed. Interestingly, the presence of both (half‐integer, half‐integer, half‐integer) and (half‐integer, integer, half‐integer) diffraction peaks in the *T*′ phase is inconsistent with the rules established by Glazer^21^ for octahedral tilts in perovskites—only (half‐integer, half‐integer, half‐integer) peaks should be observed if these peaks originated exclusively from the predicted *a* ^−^ *b* ^−^ *c* ^0^ tilt pattern. This illustrates the importance of other structural distortions within the unit cell. On the other hand, the (half‐integer, half‐integer, half‐integer) peaks for the *S*′ phase are consistent with the observed *a* ^−^ *b* ^−^ *c* ^−^ tilt pattern.

Next, we calculated the spontaneous polarization using Born effective charges and cation off‐center displacements.^22^ The diagonal elements of the Born effective charge tensor are listed in **Table** **2**. The spontaneous polarization of the *S*′ phase is 1.10 C m^−2^, which lies halfway between the *R* and *T*′ phases, polarization values of 0.98 and 1.33 C m^−2^, respectively. Previous reports^8^ have identified a strain‐induced *R* **→** *M*_A_ **→** *M*_C_ **→** *T* transition for the tetragonal *T*′‐like films. The *M*_A_ phase corresponds to an *a* ^−^ *a* ^−^ *c* ^0^ tilt pattern with *β* = 88.1 and *b/a* = 1 structural parameters, whereas the *M*_C_ phase corresponds to an *a* ^−^ *a* ^0^ *c* ^0^ tilt pattern with *β* = 90 and *b/a* = 1 structural parameters. Here, for the *T*′ phase that we have studied, the structural parameters are *β* = 88.1 and *b/a* = 0.9790 with an octahedral tilt pattern of *a* ^−^ *b* ^−^ *c* ^0^. Consequently, owing to the crystal geometry and the octahedral tilt patterns, we get (*Px* > *Py*) < *Pz* for the *T*′ phase studied. The polarization vector for the *T*′ phase (*P*_1_ space group), thus, lies along a path joining the *M*_A_ (*Cc* space group) and *M_C_* (*Pm* space group). The polarization of the *S*′ phase is commensurate with the changes in the lattice parameters (*c*/*a* ratio). Table 2 also lists the computed piezoelectric coefficients (*e*_33_) using the modern Berry‐phase approach.^23^, ^24^ The computed values are 3.85, 3.50, and 2.19 for the pure *R*, *S*′*,* and *T*′ phases, respectively. We find that there is an actual decrease in *e*_33_ as we move from *R* to *S*′ to *T*′, consistent with the experimental observation that the *T*′ phase has lowest piezoresponse.^25^

**Table 2:** The diagonal elements of the Born effective charge tensor, calculated polarization, and computed piezoelectric coefficients (*e*_33_) for the *R*, *S*′, and *T*′ phases of BiFeO_3_

Elements	*R*	*S*′	*T*′
Bi	[4.80 4.85 4.84]	[5.11 4.64 4.67]	[5.25 5.11 3.55]
Fe	[4.20 4.13 4.18]	[4.11 4.21 4.81]	[3.43 3.38 4.01]
O_1_	[−3.32 −3.46 −2.20]	[−3.25 −3.28 −2.27]	[−3.07 −2.99 −2.01]
O_2_	[−3.29 −2.20 −3.49]	[−3.54 −2.16 −3.03]	[−2.75 −2.35 −3.39]
O_3_	[−2.19 −3.45 −3.35]	[−2.41 −3.30 −3.31]	[−2.29 −2.68 −3.44]
Polarization [C m^−2^]	(0.58 0.57 0.56)	(0.58 0.52 0.80)	(0.24 0.19 1.18)
*e*_33_ [C m^−2^]	3.85	3.50	2.19

### 2.2 Electronic Structure

**Figure 2:**
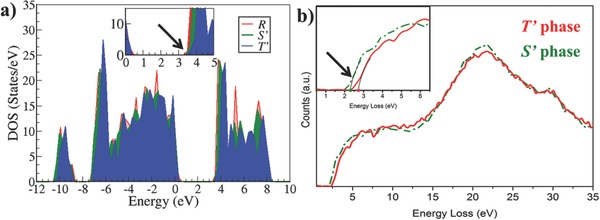
a) Total density of states for the *R*, *S*′, and *T*′ phases. The inset (arrow) highlights the onset of the conduction band and the blueshift observed, while moving from *R* to *S*′ to *T*′. b) EELS scan confirming that the band gap of *T*′ is approximately 0.35 eV larger than that of *S*′.

Crystalline BiFeO_3_ is known to be a strong antiferromagnet. The rhombohedral ground state has G‐type antiferromagnetic ordering with a calculated magnetic moment at the Fe site of 3.8 μB. For the *S*′ phase, the G‐type antiferromagnetic ordering energy is 8 meV f.u.^−1^ lower than the C‐type ordering and the magnetic moment at the Fe site is also 3.8 μB. Finally, we observe that for the *T*′ phase the G and C‐type magnetic orderings are energetically indistinguishable in agreement with elastic neutron scattering experiments that observed a coexistence of G‐type and C‐type magnetic ordering.^15^ We have observed similar behavior for fully tetragonal (*P4mm*) BiFeO_3_ at ≈7% compressive strain relative to the *R3c* rhombohedral structure.^26^ Accurate characterization of the electronic structure of BiFeO_3_ by DFT is difficult due to an inadequate description of the strong Coulomb and exchange interactions between the electrons in the *d*‐shell. For example, for the *R* phase, the LSDA+*U* approach yields *E*_g_ ≈1.9 eV,^27^ while screened exchange calculations give *E*_g_ ≈ 2.8 eV.^28^ Another report estimated *E*_g_ to be in the range of 3.0–3.6 eV using the B1‐WC and B3LYP functionals.^29^ Here, we apply the Heyd–Scuseria–Ernzerhof hybrid functional (HSE06)^30^ which is well suited for solid‐state systems and has been proven to be an accurate functional for describing the optical properties of a wide variety of semiconductors and insulators. The calculated total density of states for the *T*′, *S*′*,* and *R* phases are shown in **Figure** **2**a. The HSE06 band gaps are 3.82, 3.51, and 3.41 eV for the *T*′, *S*′, and *R* phases, respectively. The *R* phase serves as a benchmark and the calculated *E*_g_ is in good agreement with the earlier report of 3.40 eV by Stroppa et al.^31^ The *S*′ phase exhibits a blueshift by 0.1 eV with respect to the *R* phase. The band gap of the *T*′ phase is further blueshifted by 0.41 eV with respect to *R*, in excellent agreement with previous experiment, which observed a band gap increase of ≈0.4 eV.^32^

While macroscopic measurements of *E*_g_ are possible^32^ for the majority phase of the film (i.e., *T*′), this is not directly possible for *S*′. Therefore, we use electron energy loss spectroscopy (EELS) measurements in a scanning transmission electron microscope to determine *E*_g_ locally. The sample was viewed along the {100} direction, and the probe was scanned in a frame of 3 × 3 u.c. located on the center of each phase in order to reduce possible beam damage (refer to Figure S3 in the Supporting Information for EELS raw data). The same acquisition and microscopy conditions were used for both phases. In addition to the observation of fine structure distinctions at the O‐K edge and Fe‐edges in the *T*′ and *S*′ domains, which will be discussed in detail elsewhere,^33^ low loss EELS provides local band structure information that can be compared to the first‐principles results. Figure 2b displays the spectra after background subtraction and plural scattering removal. Each presented spectrum is an average of 25 spectra to improve signal‐to‐noise ratio. By fitting parabolic curves, the band gaps can be extracted as 2.39 ± 0.04 and 2.74 ± 0.03 eV for *S*′ and *T*′ spectra, respectively. Although the exact values are slightly smaller than the reported optical measurements, possibly as a consequence of instrument limitations and the sensitivity of curve fittings, the observed blueshift from *S*′ to *T*′ by 0.35 eV matches well with our DFT results and presents unique experimental information on the band structure of the *S*′ minority component.

### 2.3 Strain‐Induced Phase Transformation

**Figure 3:**
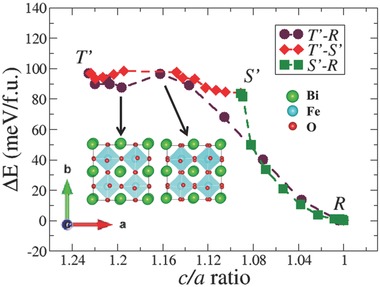
Minimum energy path characterizing *T*′*–S*′, *S*′*–R*, and *T*′*–R* phase transformations. The Bi, Fe, and oxygen atoms are shown in green, cyan, and red colors, respectively. The octahedral rotations appear along the *c*‐axis, while moving from the fourth to the fifth image as shown using the optimized structures.

To understand why a reversible phase transformation between the coexisting *T*′ and *S*′ polymorphs is possible under the application of an electric field, we now search for the possible minimum energy path (MEP) characterizing the strain‐induced phase transformations between the *T*′, *S*′, and *R* phases. For this purpose, a set of ten intermediate structures/images is constructed between the *T*′–*S*′, *S*′*–R*, and *T*′–*R* regions. Each initial image is derived from a linear interpolation between the two end members. In order to closely simulate the experimental epitaxial constraints, only the stress along the *c*‐axis is relaxed to model the MEP. We first discuss the phase transformation between the *T*′ and *R* phases. The total energy of the unit cell as a function of *c*/*a* ratio for a set of ten optimized structures connecting the *T*′ and *R* phases is shown in **Figure** **3**. Along the calculated MEP, the *T*′ phase is initially transformed into a series of triclinic structures. These optimized structures are energetically similar to the *T*′ structure and also have close *c/a* ratios even though the inplane lattice constants, *a* and *b*, are evenly spaced. Another striking feature observed here is the retention of the zig‐zag arrangement along the lattice vectors of the off‐center displacements of the Bi and Fe cations along with a similar *a* ^−^ *b* ^−^ *c* ^0^ tilt pattern. For the fifth image, a dramatic change in geometry is observed. The cations are now symmetric; simultaneously, octahedral rotations emerge about the *c*‐axis. From this point onward, the total energy of the system rapidly decreases accompanied by an increase in the magnitude of the octahedral rotations along the [111] direction, finally reaching the end point *R* phase.

To understand the strain‐stabilized coexistence of the *T*′ and *S*′ structures, we study intermediate images constructed from a linear interpolation between the *T*′ and *S*′ phases. In the *T*′*–S*′ region, we see a similar structural evolution as observed in the case of the *T*′*–R* transformation: the initial images are similar to the *T*′ phase in that they retain the zig‐zag arrangement of the Bi cations and the *a* ^−^ *b* ^−^ *c* ^0^ tilt pattern. Symmetrization of the Bi cations takes place at the fifth intermediate image, inducing rotations of the FeO_6_ octahedra about the *c*‐axis. We also observe a distinct separation between the fourth and fifth images in terms of *c/a* ratio which changes from 1.20 to 1.16 (similar to the 1.19 to 1.15 in the case of the *T*′*–R* phase transformation) despite the equal separation in the inplane, *a* and *b* lattice constants. The symmetric arrangement of Bi cations is energetically unfavorable at higher *c/a* (>1.15) ratios and there is an energy barrier of 8 meV f.u.^−1^ between these images that denotes the onset of inplane octahedral rotations. The remaining images are extremely similar to the *S*′ structure with only a 4 meV f.u.^−1^ energy difference between them, once again highlighting the flat energy landscape in the vicinity of the triclinic structures. Finally, for the *S*′*–R* region a seamless transition between the *S*′ and *R* phases is observed. The total energy of the intermediate structures progressively decreases with increasing octahedral rotations along the [111] direction. As a point of comparison, we also mapped out the MEP using solid‐state nudged elastic band (SSNEB)^34^ calculations, which remove the epitaxial constraints by relaxing the inplane lattice constants (see Supporting Information for details). The two MEPs closely follow each other for both the *T*′*–R* and *S*′*–R* phase transformations. However, the *S*′ phase is not a part of *T*′*–R* phase transformation in the SSNEB calculations but lies above the MEP as a consequence of the imposed experimental epitaxial constraints. This illustrates the importance of long‐range elastic constraints on these phases and their stability. The metastability of the *S*′ phase is further emphasized in the *T*′*–S*′ transformation where the last intermediate SSNEB images actually transform into the ground state *R* phase before reaching the end point *S*′ structure. To summarize, the flat energy landscape and the absence of a substantial energy barrier between the strain stabilized *T*′ and *S*′ phases fosters an easily reversible phase transformation between phases with and without octahedral tilts around the *c*‐axis. As these tilts are strongly coupled to lattice parameters and polarization, this leads to the high piezoelectric response across the MPB. This is in stark contrast to the transformation between *T*′ and *R* which is energetically much less favorable.

## 3 Conclusions

To summarize, we have characterized the structural, electronic, and magnetic properties of the strain stabilized *S*′ phase observed in BiFeO_3_ thin films using first‐principles calculations, synchrotron measurements, and EELS experiments. We find that the lattice parameters and polarization of this phase are intermediate to the *T*′ and *R* phases. Furthermore, the *S*′ phase is energetically close to the *T*′ phase but with octahedral rotation patterns similar to that of the *R* phase. The changes in the spontaneous polarization are approximately proportional to the changes in the lattice parameters. Both the *S*′ and *R* phases have *G*‐type antiferromagnetic ground states whereas for the *T*′ phase the G and C‐type antiferromagnetic orderings are energetically indistinguishable. A blueshift in the band gap is predicted while moving from *R* to *S*′ to *T*′, and confirmed by our EELS measurements and previous EELS measurements of the *R* phase.^30^ The study of the strain‐induced phase transformation reveals a flat energy landscape between the *T*′ and *S*′ phases, which appears to facilitate the easy movement of the phase boundary between the *S*′ and *T*′ domains under applied electrical fields and thereby leading to an enhanced piezoelectric response. These results have important implications for enhancing the materials functionality. For example, the strain‐dependent blueshift in the band gap while moving from the *R* to *S*′* to T*′ phase may lead to applications of these or related materials in photonic devices. Similarly, the knowledge of the potential energy landscape, in particular the coupling between strain and octahedral tilt modes, may lead to novel routes for designing high response piezoelectrics.

## 4 Experimental Section

*Calculations*: All calculations are based on density functional theory (DFT) with the local spin density approximation (LSDA), employing the projector‐augmented‐plane‐wave (PAW) method,^15^, ^16^ as implemented in the vienna ab initio simulation package (VASP 5.2).^14^ The PAW potentials used explicitly treated 15 valence electrons for Bi (5d^10^ 6s^2^ 6p^3^), 14 for Fe (3p^6^ 3d^6^ 4s^2^), and 6 for oxygen (2s^2^ 2p^4^). A cutoff energy of 520 eV was used to terminate the plane‐wave expansion. We considered a 2 × 2 × 2 supercell containing 40 atoms that can accommodate all the possible antiferrodistortive rotations of the FeO_6_ octahedra, while enforcing the experimental triclinic and monoclinic geometries for the *S*′ and *T*′ phases, respectively. Structural optimizations were achieved by allowing the atoms in the unit cell to relax until all the forces on each atomic site were below 5 meV Å^−1^ and simultaneously achieving a total energy convergence of 10^−6^ eV. This convergence was obtained with a 4 × 4 × 4 Monkhorst–Pack special *k*‐point grid. To correct for the metallic behavior observed in the LDA band structure, we have applied the Hubbard parameter *U* = 2 eV^27^ in our calculations. Higher values of the *U* parameter also yield similar results for the structural properties.

*Film Growth*: The experiments were performed on BiFeO_3_ films (≈45 nm thick) grown by pulsed laser deposition (PLD) on LaAlO_3_ substrates. The films were grown in a 50 mTorr oxygen background pressure, while the substrates were kept at a temperature of 675 °C. A pulsed KrF excimer laser with a wavelength of 248 nm was focused on a 10% excess Bi BiFeO_3_ sintered pellet with an energy density of 0.4 J cm^−2^ and operated at 2 Hz, resulting in an average deposition rate of ≈0.03 Å pulse^−1^.

*XRD Characterization*: Synchrotron XRD was used to determine the unit cell para­meters of the *S*′ polymorph (results for *R* and *T*′ were taken from earlier reports.^8^, ^13^ Note that the so‐called “half‐order peaks” (i.e., peaks that cannot be indexed with a unit cell containing only one formula unit) are ignored in this analysis, and therefore, by design of the analysis, this method yields the so‐called pseudocubic lattice parameters (no unit cell doubling). We know, of course, that unit cell doubling is present (otherwise, the half‐order peaks would all be absent), but the experimental data on thin films, especially on this minority phase, are insufficient for a more comprehensive analysis. For consistency with the rest of this paper's analysis, we use the notation *a*/*2, b*/*2,* and *c*/*2* for the lattice parameters obtained here. Experiments were performed at beamlines 33‐BM‐C and 33‐ID‐D of the Advanced Photon Source. Using a Pilatus 100K area detector, 3D reciprocal space maps were recorded around film peaks.^35^ The exact positions of the peaks were then determined through a 3D peak fit. An actual indexing of the film peaks is not possible based solely on inspection of the data, because this would require knowledge of the film/substrate alignment for each structural variant (domain) of the monoclinic and triclinic structures on a square lattice. The exceptions to this are the 00l peaks, which are readily indexed. To refine the lattice para­meters, we therefore created a discrete list of peak positions (Q_1_, Q_2_, …, Q*_N_*) from the diffraction maps (ignoring all information except for the Bragg angle 2*θ*). Entire families of peaks can readily be attributed to the corresponding pseudoperovskite family (for example, the peaks corresponding to 102, $\bar{1}$02, 012, 0$\bar{1}$2 are readily grouped as the {10l} family with l = 2). For each of the 4!4!4! = 13,824 permutations of the strongest peak families ({102}, {112}, {103}), a least‐square refinement to the peak positions was performed, and the permutation yielding the lowest χ_min_ was chosen to assign peaks to the list of peak positions. Keeping these assignments fixed, additional peaks were then treated similarly. Note that there are 8! = 40,320 permutations for the {h k l _fixed_} families if h ≠ k, h ≠ 0, k ≠ 0. For the refinement of the *S*′ phase, we used these peaks: 00l for l = 1,2,4; {10l} and {11l} for l = 1,2,3; {20l} for l = 2,3; and {12l} with l = 1,2. With this, we found *a*/2 = 3.91 Å, *b*/2 = 3.81 Å, *c*/2 = 4.16 Å; *α* = 90.51°, *β* = 89.38°, *γ* = 90.83°.

*EELS Characterization*: The EELS experiments were performed with a Nion UltraSTEM, operating at 100 kV accelerating voltage. EEL spectra were collected using a Gatan Enfina spectrometer, with an energy resolution of 0.5 eV for 0.1 eV/channel energy dispersion. A convergence semiangle of 30 mrad for the incident probe and the collection semiangle of 35 mrad were used for spectrum acquisitions. The spectra shown in Figure 2 were processed with the subtraction of the zero loss peak (ZLP) and the removal of a multiplural scattering. This is achieved by fitting the ZLP with a Gaussian plus Lorentzian function using Gatan Digital Micrograph software, following peak centering at zero electron volts. The band gap extractions were performed by using a parabolic curving fitting of (*E* −*E*_g_)^1/2^ to the background‐corrected band‐gap signal.^36^

## Supporting information

As a service to our authors and readers, this journal provides supporting information supplied by the authors. Such materials are peer reviewed and may be re‐organized for online delivery, but are not copy‐edited or typeset. Technical support issues arising from supporting information (other than missing files) should be addressed to the authors.

Supplementary
