# Supplementary material for: Understanding Strain‐Induced Phase Transformations in BiFeO3 Thin Films
Source: Adv Sci (Weinh). 2015 May 28;2(8):1500041. doi: 10.1002/advs.201500041 (PMC5115423; doi:10.1002/advs.201500041)
Supplement: Supplementary file 1 — Supplementary [file ADVS-2-0h-s001.pdf]

Copyright WILEY-VCH Verlag GmbH & Co. KGaA, 69469 Weinheim, Germany, 2015.

ADVANCED SCIENCE
Open Access

Supporting Information

for *Adv. Sci.*, DOI: 10.1002/advs.201500041

Understanding Strain-Induced Phase Transformations in $BiFeO_3$ Thin Films

Hemant Dixit,* Christianne Beekman, Christian M. Schlepütz, Wolter Siemons, Yongsoo Yang, Nancy Senabulya, Roy Clarke, Miaofang Chi, Hans M. Christen,* and Valentino R. Cooper*

Copyright WILEY-VCH Verlag GmbH & Co. KGaA, 69469 Weinheim, Germany, 2013.

Supporting Information

Understanding Strain-Induced Phase Transformations in $BiFeO_3$ Thin Films

Hemant Dixit, Christianne Beekman, Christian. M. Schlepütz, Wolter Siemons, Yongsoo Yang, Nancy Senabulya, Roy Clarke, Miaofang Chi, Hans M. Christen and Valentino R. Cooper

The solid-state nudged elastic band (SSNEB) calculations are performed using the VIENNA AB INITIO SIMULATION PACKAGE (VASP) Transition State Theory (VTST) code. We have used a string of ten images that are initially linearly interpolated between the initial and final state. The energy landscape characterizing *T'*- *S'*-*R* phase transitions is shown in **Figure S1**. In the case of *T'*- *S'* phase transformation, the last SSNEB images actually make a transition into the *R* phase (indicated by the arrows) before reaching the end point *S'* phase. These results clearly indicate that the *S'* phase is not a local minimum and can only be strain stabilized by the substrate in $BiFeO_3$ thin films.

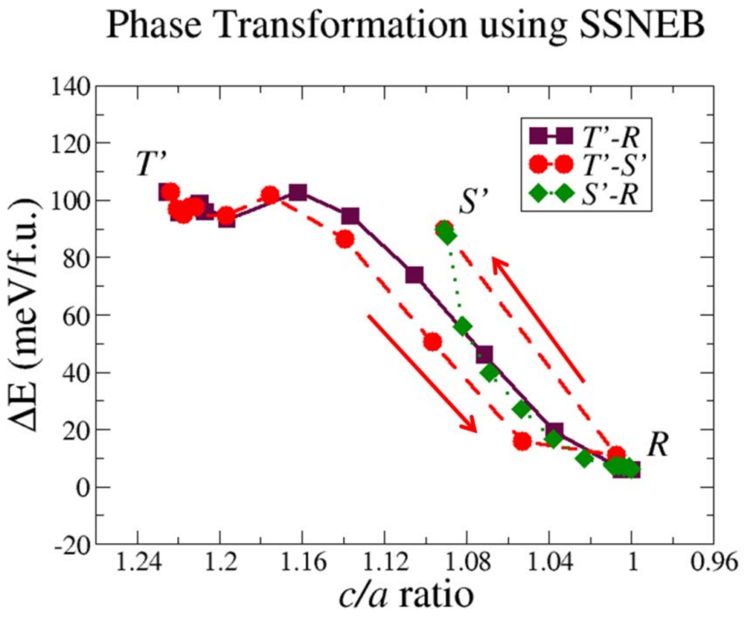

Figure. S1. (Color online) Minimum energy path characterizing *T*′-*S*′-*R* phase transformations obtained using the SSNEB method.

The X-ray diffraction (XRD) pattern for the optimized structures were calculated using the GDIS[19] software. **Figure S2** shows the calculated XRD spectrum for the *S*′ and *T*′ phases. The stronger (integer, integer, integer) peaks are much higher (~10^3) in intensity compared to the type (half-integer, half-integer, half-integer) that are present. The raw data from the calculated X-ray diffraction spectrum is compared with the experimental synchrotron XRD.

An averaged EELS spectra from *T*′ and *S*′ phases is shown in **Figure S3.** The inset picture shows a transmission electron micrograph (TEM) of the co-existing *T*′ and *S*′ phases. The EELS spectra were acquired through scanning a frame of 3×3 u.c. from the central portion of the *T*′ and *S*′ phases, in order to reduce the beam irradiation. Further, the presented EELS spectra are averaged over 25 spectra from each phase to improve signal-to-noise ratio.

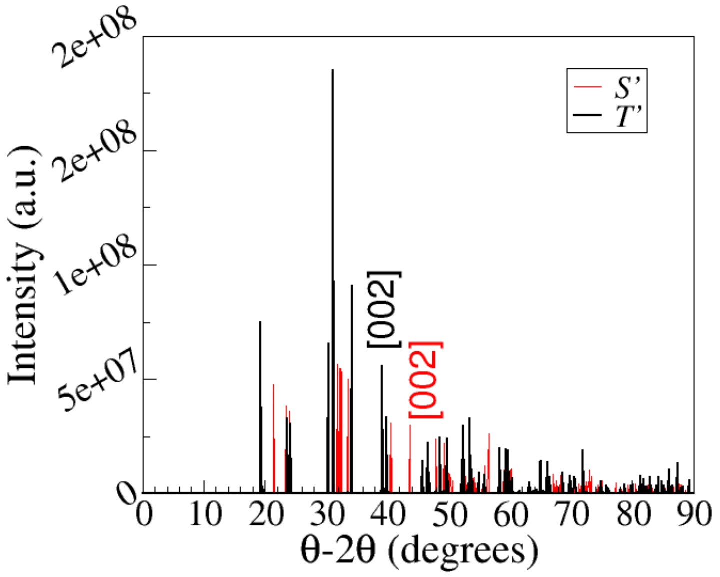

Figure. S2. (Color online) Calculated X-ray diffraction pattern for the *T′* and *S′* phases using the GDIS[19] software.

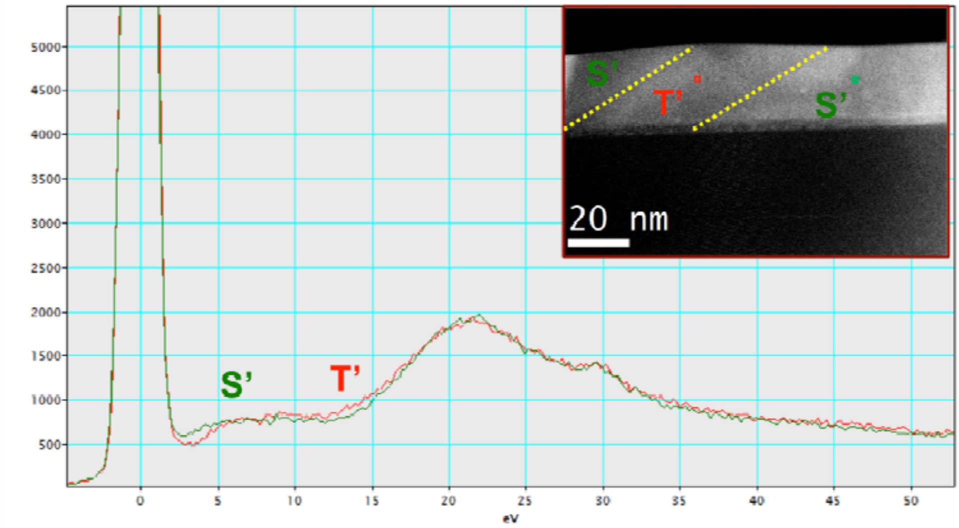

Figure. S3. (Color online) Averaged EELS spectra from *T′* and *S′* phases. These were acquired from the central area of *T′* and *S′* phases, as highlighted using the small red and cyan colored squares (representing the area of scan) through the inset image.

The optimized structures (40 atom cell) corresponding to the *T′* and *S′* phases are provided in '*cif*' format.
